# On the physiological and cellular homeostasis of ascorbate

**DOI:** 10.1186/s11658-020-00223-y

**Published:** 2020-05-29

**Authors:** Magdalena Przybyło, Marek Langner

**Affiliations:** 1 Faculty of Biomedical Engineering, Wrocław University of Sciences and Technology, 50-370 Wrocław, Poland; 2 Lipid Systems Ltd, Krzemieniecka 48C, 54-613 Wrocław, Poland

**Keywords:** Vitamin C, Biodistribution, Membrane transport, Homeostasis, Membrane potentials

## Abstract

Recent interest in the role of ascorbate in crucial metabolic processes is driven by the growing number of medical reports that show beneficial effects of ascorbate supplementation for maintaining general well-being and recovery from a variety of medical conditions. The effect of ascorbate on the local body environment highly depends on its local concentration; at low concentrations it can cause the reduction of reactive oxygen and facilitate activities of enzymes, while at high concentrations it generates free radicals by reducing ferric ions. Ascorbate serving as an electron donor assists the iron-containing proteins and the iron transfer between various aqueous compartments. These functions require effective and adjustable mechanisms responsible for ascorbate biodistribution. In the paper we propose a new biophysical model of ascorbate redistribution between various aqueous body compartments. It combines recent experimental evidence regarding the ability of ascorbate to cross the lipid bilayer by unassisted diffusion, with active transport by well-characterized sodium vitamin C transporter (SVCT) membrane proteins. In the model, the intracellular concentration of ascorbate is maintained by the balance of two opposing fluxes: fast active and slow passive transport. The model provides a mechanistic understanding of ascorbate flux across the epidermal barrier in the gut as well as the role of astrocytes in ascorbate recycling in the brain. In addition, ascorbate passive diffusion across biological membranes, which depends on membrane electric potentials and pH gradients, provides the rationale for the correlation between ascorbate distribution and the transfer of iron ions inside a cell. The proposed approach provides, for the first time, a mechanistic account of processes leading to ascorbate physiological and cellular distribution, which helps to explain numerous experimental and clinical observations.

**This article was specially invited by the editors and represents work by leading researchers**.

## Introduction

Vitamin C (ascorbate) is a compound indispensable for maintenance of redox homeostasis in aqueous body compartments [1, 2]. Initially, it was considered as one of many antioxidants needed for the control of the redox potential and the level of reactive oxygen species (ROS) [3]. Later it was observed that ascorbate at high concentrations (above 1 mM) is toxic to cancer cells, revealing the complex nature of ascorbate functions. The importance of ascorbate homeostasis has been further strengthened by the discovery that there are two dedicated ascorbate transporters, namely SVCT1 and SVCT2, encoded in genes SLC23A1 and SLC23A2 [4, 5]. The two discoveries shed a new light on the essential role of ascorbate in cellular processes, other than being simply one of many antioxidants [2]. During the last decade a number of ascorbate-dependent enzymes have been identified [6, 7]. It has also been observed that ascorbate is required for maintenance of the oxidation level of transition metals, important functional components of diverse metabolic processes [8]. Handling of transition metals by cells requires fine-tuned mechanisms, which allow the accumulation of transition metals (Fe and Cu) for biochemical reactions, yet reducing the risk of toxic effects caused by their labile forms [8]. Ascorbate, being an electron donor, can function both as an antioxidant and pro-oxidant [1]. At low concentrations, it reduces reactive oxygen species (ROS), which are generated as a result of metabolic activities and/or an exposure to exogenous hazards [2, 3]. However, at high concentrations it can act as a pro-oxidant by reducing transition metals (iron, copper). Reduced transition metals react with hydrogen peroxide, leading to the formation of highly reactive and damaging hydroxyl radicals, via the Fenton reaction [8–11]. The effect of ascorbate on iron is not limited to aqueous solutions but may also affect electron transfer in iron containing proteins, which are involved in: oxygen storage and transport (hemoglobin and myoglobin), oxygen sensing and hypoxic regulation (HIF prolyl hydrogenases), energy production (cytochrome c, cytochrome c oxidase and NADH dehydrogenase), intermediary metabolism and detoxification (ribonucleotide reductase, amino acid oxidases, fatty acid desaturases, cytochrome P450 and catalase), synthesis of hormones and neurotransmitters (tryptophan hydroxylase, tyrosine hydroxylase and thyroperoxidase) as well as playing a role in host defense and inflammation (myeloperoxidase, NADPH oxidase, indoleamine 2,3-dioxygenase, nitric oxide synthesis and lipoxygenases) [10, 12]. Iron is also an indispensable element of proteins coordinating and regulating the expression of genetic material [13–15].

Iron-containing proteins are distributed between different aqueous body compartments, where levels of redox potential need to be adjusted to a specific value ensuring the optimal conditions for iron homeostasis and functioning of iron-containing proteins [16]. The local homeostasis of iron requires maintenance of the redox potential, access to labile ions and suppression of any potential damage associated with generation of ROS [17, 18]. The biologically relevant iron ions are water soluble Fe^2+^ (negative logP) and water insoluble Fe^3+^ (high and positive logP) [12]. The dramatic difference of their water solubility is utilized by biological systems to facilitate effective iron biodistribution, simultaneously limiting the risk of systemic toxicity. This is achieved by the coordinated set of proteins, which transfer, store and exchange iron ions in specific, with respect to redox potential, aqueous environments. The iron homeostasis requires effective storage and distribution systems, which are based on interconnected reduction and oxidation processes mediated by dedicated proteins, including reductases, carriers (transferrin), storage complexes (ferritin) and membrane transporters and receptors (divalent metal ion transporters or ferroportin) [12]. Each element of the system requires specific aqueous phase redox potential, which can be provided by coordinated fluxes of a hydrophilic, readily accessible electron-rich compound [8]. Such a compound is effective in the complex topology of a biological system only when its local availability can be tuned to a specific concentration. A number of experimental studies have demonstrated that ascorbate is such a compound [11, 18]. The ability of ascorbate to reduce the ferric ion (Fe^3+^) to the ferrous ion (Fe^2+^) enhances non-heme Fe^3+^ absorption from the diet. Reduction of Fe^3+^ to Fe^2+^ is the first and necessary step for entering a cell through a divalent metal ion channel [19]. Ascorbate is also required by a wide range of Fe^2+^/ *a*-KGDDs enzymes dispersed among different cellular aqueous compartments [16]. For example, the Jumonji C (JmjC) domain-containing histone demethylases (JHDMs), DNA demethylase of the AlkB homolog (ALKBH) family, and the ten-eleven translocation (TET) family of DNA hydroxylases are located in the nucleus [13–15]. It has been shown that in the cytoplasm ascorbate stimulates the synthesis of the cellular iron storage protein, ferritin, by inhibiting its lysosomal degradation and cellular iron efflux [11]. In the cytoplasm ascorbate is also necessary for the activity of collagen prolyl hydroxylases, a family of Fe^2+^/ *a*-KGDDs that regulate collagen synthesis [20].

Fe^2+^/ *a*-KGDDs have relatively high Km values for ascorbate (the concentration required for half-maximal reaction rates is 140–300 *μ* M). The low affinity for ascorbate requires above 1 mM ascorbate intracellular levels for optimal catalytic activity, and it cannot be substituted by other antioxidants, indicating a specific need for ascorbate as a cofactor for these enzymes [15, 21]. This value is much higher than the serum ascorbate concentration (about 50 *μ* M). Therefore, the barrier between interstitial and intracellular aqueous phases (plasma membrane) should facilitate and maintain the high ascorbate concentration gradient. In addition, there are large differences between intracellular ascorbate concentration gradients generated across plasma membranes, which can range from 0 in erythrocytes to 10 mM in neurons [2]. Such complex spatial ascorbate distribution requires a transport system which is based on opposing fluxes at both physiological and cellular levels. Despite abundant evidence showing that ascorbate is an important regulator of metabolic and genetic processes, there is no good understanding of the molecular processes leading to its biodistribution [8]. There are two dramatic examples where the protein-based ascorbate flow between aqueous compartments is inadequate. Epithelial cells, which extract both ascorbic acid and dehydroascorbic acid (DHA) from the gastrointestinal tract (GIT), have SVCT1 transporters on the apical side. They also have SVCT2 transporters on the basolateral side. This indicates that they extract ascorbate from both the gastrointestinal tract and interstitial fluids [22]. Consequently, there is no effective mechanism ensuring ascorbate flux from the gastrointestinal tract to the blood circulation. DHA handling by astrocytes in the brain tissue is the other example. Astrocytes reduce DHA produced by neurons. Whereas GLUT-base fluxes of DHA are well understood, the release mechanism of ascorbate from astrocytes to interstitial fluid remains unknown [23]. The ascorbate intracellular distribution is even less understood, and, with the exception of mitochondria, has never been studied [24]. In order to resolve all issues related to ascorbate distribution between various body aqueous compartments, a consistent model, containing ascorbate fluxes in and out of each aqueous compartment, is needed.

Until recently, it had been assumed that the hydrophilic ascorbate (logP = − 1.85) is not able to pass unassisted across the hydrophobic lipid core of the biological membrane. Consequently, it has been assumed that a local level of ascorbate is facilitated exclusively by membrane proteins. Sodium vitamin C transporters (SVCTs) for ascorbate and GLUT channels for DHA contribute to ascorbate homeostasis [25]. However, the combination of ascorbate intake by cells (SVCT transporters) and bi-directional transport of dehydroascorbate (GLUTs) is not sufficient to construct an effective model for the ascorbate homeostasis in various body or cellular compartments. In order to resolve the difficulty, the transport and intracellular reduction of DHA have been postulated as a mechanism responsible for ascorbate homeostasis, at least in some situations. For example, the presence of ascorbate in erythrocytes, which do not have SVCT transporters, has been explained by the reduction of DHA entering the cells via GLUT channels [26]. However, there are a number of arguments which render the role of DHA transport in ascorbate homeostasis unlikely. DHA is toxic, and hence it is efficiently reduced to ascorbate inside respective cells [27]. The unrestricted bidirectional transport of DHA prevents the formation of any DHA concentration gradients. Consequently, its concentration in major aqueous compartments should not exceed the value of 1–2 μM, an insignificant quantity when compared with concentrations of ascorbate, which range from 50 μM to 10 mM [27]. The main difficulty with the current understanding of ascorbate homeostasis is caused by the assumption that only protein-based transport is feasible. This approach seems to be sufficient to explain the ascorbate level in cells in culture, where the active transport is balanced mainly by the ascorbate metabolic consumption and oxidation subsequently followed by DHA release to the medium [28]. However, at the physiological level, such simplification is not practical. As pointed out earlier, there are important cases where the mechanism of ascorbate redistribution cannot be understood exclusively in terms of transport through known membrane proteins. The main problem with the protein-based model is that there is no known mechanism which enables ascorbate flow out of a cell.

Recently, it was demonstrated, and later confirmed, that ascorbate crosses the biological membrane by passive diffusion through the lipid bilayer [29, 30]. The finding provides the missing element for the construction of an effective conceptual model describing the spatio-temporal ascorbate distribution between body aqueous compartments. The ascorbate passive diffusion across the biological membrane can be reliably approximated with lipid bilayer-based experimental model systems. The approximation is routinely and successfully used in pharmacological sciences ([31, 32] and citations therein).

The passive transport of a weak acid (ascorbic acid, pK_1_ = 4.2 and pK_1_ = 11.6 [1]) through the lipid bilayer of biological membrane depends, in addition to the concentration gradient, on the membrane electrical potential difference and pH gradients between adjacent aqueous compartments [33]. Considering properties of membrane barriers and aqueous phases the details of ascorbate distribution within a single cell can be proposed. An approach similar to that proposed by Scott et al. [33], for the prediction of the intracellular distribution of charged amphiphilic compounds (− 1 < logP < 4), can be readily adopted to evaluate the correlation between the spatial distribution of ascorbate and processes requiring transfer of electrons as described by D’Anielo et al. [6].

At physiological pH, ascorbate is predominantly in the monoanionic form. Products of its oxidation are at low concentrations in vivo, due to their inherent instability and/or rapid elimination by glutathione (GSH)- and NADP-dependent enzymatic and non-enzymatic reactions [1, 34]. Consequently, in physiological conditions, local ascorbate homeostasis can be approximated by its reduced mono-anionic form. In some cases, however, the dehydroascorbate flux needs to be accounted for, as demonstrated by a model describing the ascorbate homeostasis in the brain [35].

The presented model of ascorbate homeostasis is based on the following assumptions: the steady state homeostasis of ascorbate can be quantitated only by the ascorbate mono-ion. The ascorbate metabolites in general and DHA in particular are short-lived. Consequently, their concentrations do not exceed the value of 5% of the ascorbate and are rapidly equilibrated between various aqueous compartments due to facilitated diffusion. The ascorbate passive transfer across the lipid bilayer is slower than transfer by transporters. Specifically, the SVCT2-based active transport in human melanoma cells (SK-MEL-131) generates the ascorbate flux characterized by V_max_ of 150 pmol/min/million cells [36]. The estimated ascorbate passive diffusion for the cell system gives values about two orders of magnitude smaller, without accounting for plasma membrane electric potential. Consequently, the ascorbate level inside the cell is maintained by the fast and efficient active transport, the metabolic consumption, effective elimination of metabolites by reduction mechanisms and/or their release to the interstitial fluids and slow passive diffusion of ascorbate. Such an arrangement results in situations where steady-state ascorbate concentration depends on the two fluxes and the metabolic consumption (Eq. 3). The energetic cost of such a system is relatively low, due to the very low value of the lipid bilayer permeability coefficient for ascorbate (10^− 8^ cm^2^/s). The passive diffusion out of the cell is aided by the membrane electric potential (Table 1), which can elevate the effective flux by an order of magnitude for differentiated cells [43].

**Table 1:** Electrical membrane potentials and pH for selected organelles and their effect on the local ascorbate concentration assuming that its concentration in the cytoplasm equals 1 mM [6, 33, 37–42]

Membrane	Membrane potential	pH	${\boldsymbol{C}}_{\boldsymbol{asc}}^{\boldsymbol{el}}\left[\boldsymbol{mM}\right]$^**a**^	${\boldsymbol{C}}_{\boldsymbol{asc}}^{\boldsymbol{pH}}\left[\boldsymbol{mM}\right]$^**a**^
Lysosome	+ 20 mV	5	2.2	0,006
Endosome	+ 70 mV	7	9	1
Mitochondria	(−150 mV) - (− 180 mV)	8	0.003	6.3
Golgi	+ 30 mV	6–6.7	3.2	0,2
Nucleus	+ 3 0 mV	7.2	3.2	1

^a^*The hypothetical ascorbate concentration was calculated for a single factor pH or electrical membrane potential,*${C}_{asc}^{pH}\left[ mM\right]\ \mathrm{and}\ {C}_{asc}^{el}\left[ mM\right]$*, respectively*

Using the model, the overall ascorbate balance at the physiological level can be accurately described. In addition, the dependence of ascorbate distribution across cellular membranes on electrostatic potential and/or pH gradients can be accounted for. This allows one to propose the ascorbate distribution within a cell and subsequent correlation with redox potentials, as required for proper functioning of transition metal containing enzymes as well as transfer of metal ions themselves in the cytoplasm. Finally, the model was used to provide a rationale for the effect of ascorbate on selected medical conditions.

### Physiological homeostasis of vitamin C

**Fig. 1:**
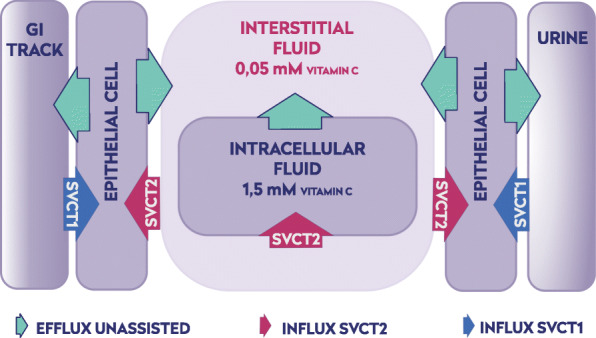
Spatial distribution of plasma membranes containing SVCT1 or SVCT2 with respect to adjacent aqueous phases. Plasma membranes containing SVCT1 separate the cytoplasm of epithelial cells from the outer aqueous phase (GIT volume, urine). Plasma membrane containing SVCT2 separates cytoplasm of a cell from the interstitial fluid. Consequently, epithelial cells contain both SVCT1 and SVCT2 transporters, whereas all other cells are practically devoid of SVCT1

In most mammals, the temporal whole-body balance of ascorbate is tightly controlled by opposing ascorbate fluxes, namely, endogenous synthesis, intake from diet, metabolic consumption and excretion. The temporal variation of the ascorbate concentration from the physiological level in serum $\frac{\mathrm{d}A{(t)}_{tot}}{dt}$ can be described with the following equation:

1 $$ \frac{\mathrm{d}A{(t)}_{tot}}{dt}=J{(t)}_{met}+J{(t)}_{out}+J{(t)}_{endogenous}+J{(t)}_{diet} $$

where *J*(*t*)_*met*_ is the ascorbate loss due to the metabolic consumption, *J*(*t*)_*out*_ is the ascorbate excreted from the organism (kidney, digestive tract, sweat, etc.), *J*(*t*)_*endogenous*_ and *J*(*t*)_*diet*_ represent the ascorbate synthesized in the body and absorbed from the diet, respectively. The ascorbate homeostasis in most animals is maintained by the feedback mechanism between the level of the endogenous supply and the temporal physiological concentration in interstitial fluids. In humans, however, this feedback mechanism is broken, due to the mutation in the L-gulono- *γ*-lactone oxidase (GULO) gene, thus making vitamin C an essential dietary component [2, 44, 45]. Consequently, the supply of ascorbate from the diet is neither efficient nor effectively adjusted to the temporal physiological requirements. The dysfunctionality of the ascorbate homeostatic feedback results in deficiencies, either transient or permanent [46, 47]. When adequately supplied, ascorbate level in serum varies within the narrow concentration range of 50–80 *μ* M [2]. At the physiological level, ascorbate and its oxidized form (dehydroascorbate) are absorbed or secreted by luminal cells in the gastrointestinal tract by passive diffusion, facilitated diffusion, and active transport [20, 48]. To model the ascorbate distribution within the body, a conceptual system of aqueous compartments, separated by biological barriers, can be constructed (Fig. 1). The quantity of ascorbate available from the gastrointestinal tract volume, C_GI_(t), depends exclusively on the diet and the intestinal absorption. The low-affinity, high-capacity SVCT1s are located on the apical side of luminal cells in GIT or proximal tubules in the kidney. They extract and accumulate ascorbate from outer aqueous phases (food or urine) in their cytoplasm [20]. Concentration of ascorbate in these cells reaches the level of 1.5 mM, enabling the membrane potential-assisted passive transport of ascorbate to the interstitial fluids and/or back to the GIT [2]. There are no known proteins that would feed ascorbate to interstitial fluids from the epithelial cells. The water-soluble ascorbate molecules have free access to most extracellular compartments through leaky vasculature; therefore its concentration throughout the body, for all practical reasons, can be treated as uniform. There is however an important exception – the brain, where autonomous interstitial fluid is surrounded by tightly packed endothelial barriers [23, 35, 49]. This allows the ascorbate concentration in interstitial fluids of the brain to be maintained at the level of 0.5 mM [50].

**Fig. 2:**
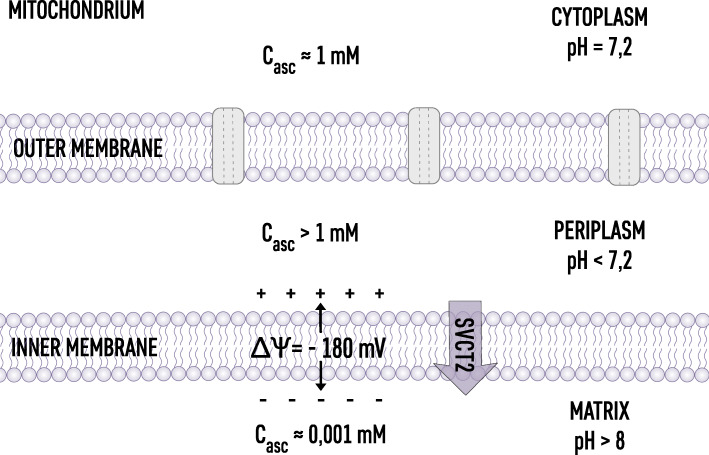
Ascorbate distribution in mitochondria. In the cytoplasm where pH = 7.2 ascorbate concentration is maintained by SVCT transporters in the plasma membrane. In the periplasm, where pH < 7.2, high proton concentration will produce C_asc_ > 1 mM. In the mitochondria matrix the ascorbate concentration will be effectively suppressed by the highly negative electrostatic potential across inner mitochondria membranes. Mitochondria contain SVCT2, indicating that the ascorbate distribution is rigorously maintained

Ascorbate is extracted from interstitial fluids by cells into their cytoplasm by high affinity/low capacity SVCT2 transporters [20, 51–53]. The quantity of intracellular ascorbate is individually adjusted to a temporal level of metabolic activity by membrane transporters, whose number is epigenetically controlled [53, 54]. Consequently, the level of ascorbate concentration inside cells may differ widely from 0.05 mM in erythrocytes, whose plasma membrane lacks SVCT transporters, to as high as 10 mM in neurons [2]. Such complex ascorbate concentration arrangement implies that not only the quantity but also the distribution of ascorbate is of fundamental importance for the survival, as dramatically demonstrated by an experiment with SVCT2 knock-out mice, which died immediately after births [55].

### Significance of ascorbate passive diffusion through the biological membrane

The maintenance of spatial distribution of ascorbate requires fluxes which need to be continuously adjusted to temporal metabolic requirements [20]. In the model, the accumulation of ascorbate inside cellular cytoplasm by SVCT transporters is balanced by passive diffusion, which is proportional to the concentration gradient according to the Fick law [33] and membrane potential. At the steady state ascorbate fluxes through the plasma membrane can be described by Eq. 2:

2 $$ {J}_{active}-{J}_{passive}={n}_{SVCT1}{a}_{SVCT1}{C}_{out}+{n}_{SVCT2}{a}_{SVCT2}{C}_{out}-{J}_{passive}=0 $$

The equation shows that the intracellular ascorbate concentration is generated by a specified number of SVCT transporters present in the plasma membrane. The low value of the ascorbate membrane permeability constant (10^− 8^ cm/s [29]) may indicate that a relatively small number of SVCT transporters is required for maintenance of the ascorbate intracellular level. A specific SVCT location on the plasma membrane is dictated by the type of adjacent aqueous phase. The plasma membrane on the apical side (volume of gastrointestinal tract or urine) contains exclusively SVCT1 transporters. They are characterized by low affinity (K_m_ ≈65 *μM* − 252 *μM*) and high capacity (${V}_{max}=15\frac{pmol}{\mathit{\min}}/ cell$), ensuring large fluxes of ascorbate at the high concentration regime. Such arrangement enables a high ascorbate concentration gradient to be maintained. When plasma membrane separates cytoplasm from the interstitial fluid it contains only SVCT2 transporters. SVCT2 transporters are characterized by high affinity to ascorbate (K_m_ ≈8 *μM* − 69 *μM*) and low capacity (${V}_{max}=1\frac{pmol}{\mathit{\min}}/ cell$) [56, 57]. Therefore, the transporter is able to extract ascorbate from interstitial fluids, where its concentration is typically low (*C* = 10 *μM* − 80*μM*). A schematic presentation of SVCT transporter distribution between various parts of plasma membranes is shown on Fig. 1. The plasma membrane of epithelial cells faces simultaneously the outer and interstitial aqueous phases. The spatial separation of SVCT1 and SVCT2 to the relevant apical and basal fraction of plasma membranes is a telling confirmation of the model predictive capability [20, 53]. The architecture of fluxes envisioned by the new model, where active transport is combined with passive diffusion, allows for the following predictions. First, the ascorbate is excreted not only by the kidney but also via the gastrointestinal tract. Excessive ascorbate concentration in cytoplasm of epithelial cells induces passive diffusion flow back to the GIT. The prediction is supported by the observation that the fraction of digested vitamin C dramatically decreases with its dose [2, 58]. This mechanism also explains the rapid elimination of ascorbate following its intravenous injection in a high quantity (50 g or more) [58]. Secondly, the physiological arrangement of ascorbate transporters ensures its low interstitial ascorbate concentration, thus limiting the risk of generation of a significant quantity of toxic labile transition metals [59, 60]. The ascorbate homeostasis in interstitial fluids (deficient in humans) provides a uniform baseline against which all other cells adjust their cytoplasmic concentrations by simply controlling the number of SVCT2 transporters [50]. Therefore, the ascorbate deficiency will affect interstitial compartments first. The cellular supply can be corrected by elevated expression of ascorbate transporters. It has been demonstrated that ascorbate deficiency promotes upregulation of both SVCT1 and SVCT2 mRNA expression in various tissues [54, 61].

### Ascorbate homeostasis inside the cell

As has been pointed out, the quantity of ascorbate in the cytoplasm is regulated by the number of SVCT transporters and homeostasis in interstitial fluids. However, the eukaryotic cell is a complex maze of aqueous phases separated by biological membranes differing in composition and properties [62]. Consequently, it can be hypothesized that the local ascorbate concentration may depend on a specific aqueous compartment. This is significant since inside the cell, there are a number of aqueous compartments with processes requiring different levels of redox potential [17, 63–66]. This variation is especially relevant when iron homeostasis is considered [8, 12, 67, 68].

Since there are not many endo-membranes containing ascorbate transporters (mitochondria being an exception [24]), passive diffusion is the only mechanism able to generate ascorbate concentration gradients inside the cytoplasm. As discussed elsewhere, the passive transport of weak acid through a biological membrane will generate a concentration gradient difference between separate aqueous compartments, which will depend on the membrane electric potential and pH gradients [33]. Electric potentials are generated across the plasma membrane, some endo-membranes, nuclear membrane or inner membrane of mitochondria by electrogenic processes, whereas the local pH value depends on proton transport mechanisms [37].

The endosome may serve as an example for the demonstration of how changing membrane and entrapped aqueous phase properties may correlate with distribution of ascorbate and redox potential-dependent metabolic processes. Transferrin-triggered endocytosis is a mechanism involved in the iron intake from interstitial fluid. In the interstitial fluid Fe^3+^ ions are firmly bound to transferrin. The transferrin containing iron ions is internalized by a cell after its attachment to transferrin receptor 1 (TfR1) followed by the internalization of the whole complex via receptor-mediated endocytosis [69]. In the endosome the iron is reduced to the Fe^2+^ form and dissociates from transferrin. The reduced iron ion is transferred to the cytoplasm through divalent metal transporter 1 (DMT1) [22]. The high concentration of ascorbate in endosomes will support the process [8]. But in cytoplasm the large labile iron pool will expose the cell to the risk of damaging oxidation [10, 60, 70]. Therefore, the redox potential in the cytoplasm should be maintained at the level which will facilitate the iron redistribution to selected compartments (nucleus, Golgi or mitochondria) or stored within ferritins [8]. Electrical membrane potentials and pH gradients across cellular membranes will affect the distribution of charged amphiphilic compounds throughout the cell volume [33]. Table 1 shows electric membrane potentials and pH of the encapsulated aqueous phases of selected organelles along with calculated ascorbate concentrations (in equilibrium). Ascorbate concentration in the cytoplasm is higher (above 1 mM) by more than an order of magnitude than that in interstitial fluids (0.05 mM) [2]. Consequently, ascorbate concentration can be elevated or depressed relative to the cytoplasmic value.

3 $$ {n}_{SVCT1}{a}_{SVCT1}{C}_{out}+{n}_{SVCT2}{a}_{SVCT2}{C}_{out}= PA\left({C}_{in}-{C}_{out}\right)+{a}_{met}(t) $$

The transmembrane distribution of permeable ions (ascorbate) in equilibrium or steady state can be quantitated with the Nernst formula [33]:where ${Asc}_{out}^{-}$ refers to the ascorbate concentration in the interstitial fluid in the case of the plasma membrane or cytoplasm in the case of an organelle. ${Asc}_{in}^{-}$ refers to ascorbate concentration in the cytoplasm in the case of the plasma membrane or inner organelle aqueous phase for endo-membranes. The steady-state ascorbate concentration distribution across a membrane can be therefore quantitated by the following equation:

4 $$ {Asc}_{in}^{-}={Asc}_{ou\mathrm{t}}^{-}\exp \left(\frac{zF}{RT}\Delta  \varphi \right) $$

$$ where\ {C}_{in}={C}_{out}\exp \left(\frac{zF}{RT}\Delta  \varphi \right) $$

The equation shows that the intracellular ascorbate level is regulated by a number of SVCT transporters (*n*_*SVCT*1_ and *n*_*SVCT*2_), the plasma membrane electrical potential difference, *∆φ* and temporal metabolic activity *a*_*met*_(*t*). The number of ascorbate transporters is epigenetically controlled, whereas the plasma membrane potential is independently maintained by electrogenic processes [38]. For example, the electrical potential of erythrocyte plasma membrane is lower than − 10 mV (negative inside) and there are no SVCT transporters; consequently the intracellular concentration of ascorbate does not differ from that of the blood plasma [26, 71]. In many other cells the electrical potential of the plasma membrane is negative inside and affects the ascorbate balance (Eq. 3). The ascorbate passive diffusion-based model provides a rationale for the higher sensitivity of cancer cells to the elevated ascorbate level in cell cultures and in vivo. The electric plasma membrane potential may play a role since in cancer cells it is significantly lower (from – 5 mV to – 20 mV) than that in normal cells (from – 50 mV to – 90 mV) [4, 43, 72]. Specifically, the ascorbate concentration gradient across the plasma membrane at steady state will be affected by active transport towards the cytoplasm, and passive diffusion in the opposite direction, which is enhanced by the negative inside membrane electric potential. The lowering of the plasma membrane electric potential, while all other parameters remain unchanged, will result in significant elevation of the cytoplasmic ascorbate concentration (by at least a few mM). Consequently, in cancer cells the intracellular ascorbate level may exceed the concentration required for triggering the release of labile iron ions, which will generate free radicals causing cell death. This is in good agreement with data presented by Uetaki et al. [73], who suggested that vitamin C in high doses promoted cancer cell death by inhibiting energy metabolism via NAD depletion, induced by H_2_O_2_ generation.

The effect of local pH on ascorbate concentration in a specific cellular compartment is variable [74]. The local pH depends mainly on proton transporters [75, 76]. Concentrations of protonated [HA] and deprotonated [A^−^] molecules of weak acid with a characteristic pKa are described by the Henderson-Hasselbalch relationship:

$$ \left[{A}^{-}\right]=\frac{10^{pH}}{10^{pH}+{10}^{p{K}_a}}C\kern0.5em \left[ HA\right]=\frac{10^{pK_a}}{10^{pH}+{10}^{p{K}_a}}C $$

where its concentration equals C. At the steady state the relative concentrations of a weak acid (ascorbate mono-ion) in aqueous compartments separated by biological membrane and with different pH can be quantitated using the following formula [74]:

$$ \frac{{\left[{H}^{+}\right]}_{out}}{{\left[{H}^{+}\right]}_{in}}=\frac{{\left[{A}^{-}\right]}_{in}}{{\left[{A}^{-}\right]}_{out}} $$

where [H^+^]_out_ and [H^+^]_in_ are the proton concentration outside and inside, respectively. Table 1 shows that the [H^+^] may vary by two orders of magnitude between various intracellular aqueous compartments. Such large differences will have an effect on ascorbate distribution [33].

The arguments listed above show that, in order to describe the ascorbate spatial distribution correctly, a qualitative model is required. The model assumes that the active transport, facilitated by the dedicated ascorbate transporters in the plasma membrane, will generate a high ascorbate concentration in the cytoplasm. The intracellular ascorbate distribution between various organelles is facilitated by membrane electrical potentials and pH of entrapped aqueous phases. The presented model describes ascorbate distributed within the cell cytoplasm demonstrating the role of electrical potentials of endo-membranes and local pH. Consequently, there are regions within the cytoplasm where the ascorbate concentration is elevated (early endosomes and nucleus) or depressed (mitochondria and lysozymes). The ascorbate concentration in a compartment will affect its redox potential and when combined with the presence of transition metal ion (iron), oxidizing or reducing conditions will be created. Transition metals are present throughout the cell volume; therefore they need to be maintained at a specific oxidation level so that excessive damage, due to the generation of free radicals, will not occur [18, 69]. The ascorbate is required for processes involving iron reduction and/or electron transfer facilitated by Fe^2+^/ *a*-KGDDs enzymes [8]. The effective execution of metabolic processes with participation of transition metal ions requires compartmentation so a specific redox potential will be locally maintained [1].

As demonstrated in Table 1, the ascorbate distribution within the cell can be arbitrarily divided into three distinct regions: the cytoplasm, where the ascorbate concentration is controlled predominantly by plasma membrane SVCT transporters [2]; regions with depressed ascorbate concentration down to a micromolar level (oxidative environment); and regions with elevated concentrations (reducing environment) relative to the cytoplasm. For example, in the nucleus and Golgi, where metabolic activity of Fe^2+^/ *a*-KGDDs enzymes is high, the electric potentials of the membrane (positive inside) elevates the level of ascorbate. In mitochondria, the ascorbate level is suppressed by the highly negative inner-membrane electric potential (typically higher than – 150 mV [77]).

The vesicular transport is likely to be accompanied by the evolving properties of the entrapped aqueous phase with respect to the ascorbate and pH level [78]. In the early endosome, the positive potential inside (+ 50 mV – + 90 mV) and neutral pH will generate the flow of ascorbate from the cytoplasm, increasing the local reducing capacity. The rise of ascorbate concentration will generate labile transition metals, making them ready for transfer by DMT1 to the cytoplasmic side for storage and redistribution [68]. In late endosomes, following association with lysozyme, where the membrane potential increases to + 20 mV, with a simultaneous pH drop to 5, the depleted ascorbate will create an environment suitable for oxidation [79]. In this case, the ascorbate passive flow is affected by the changing membrane potential, whereas the pH level is maintained by proton pumps [37].

The other compartment where the ascorbate level is expected to be elevated is the nucleus. The nuclear membrane has a positive electric potential inside (+ 30 mV) and neutral pH. The activity of Fe^2+^/ *a*-KGDDs enzymes, critical for the structuring and effective decoding of genetic information, requires the presence of a reducing agent sufficient for sustaining the activity of enzymes and acting as an antioxidant, protecting the integrity of genetic material.

The aqueous space in mitochondria is the location of electron transfer processes mediated by water soluble compounds and compounds located at the membrane surfaces (NADH, FAD). Therefore, the tight control of redox potential in the mitochondria matrix aqueous phase is of fundamental importance [80]. The highly negative electrical potential on the inner side of the inner mitochondrial membrane (− 180 mM [19, 37]) indicates that indeed the ascorbate concentration in the matrix is depressed down to the single micromolar level. Interestingly, mitochondria are equipped with SVCT2 transporters, which indicates that there is a specific level of ascorbate required for maintaining the optimal redox potential [24, 81]. The intermembrane space is different; the positive electric potential indicates that the ascorbate accumulates at the outer surface of the inner membrane, forming a barrier to free radicals generated in the oxidative phosphorylation process and controlling the redox signaling [63]. The ascorbate distribution inside mitochondria is schematically shown in Fig. 2. 

The examples presented above show that, as predicted by the model, ascorbate concentration pattern correlates with redox potential levels required by iron-containing enzymes [8].

### Medical significance of the model

Non-obvious, but experimentally demonstrated, ascorbate passive diffusion through the lipid bilayer when combined with protein-based mediated and active transports allows one to construct a model which resolves all physiologically important issues related to ascorbate homeostasis and its role in controlling metabolic processes. Application of the model allows one to account for a number of observations which have not been explained based on the protein-only approach. These include questions concerning the concentration of ascorbate in the erythrocytes, the mechanism by which astrocytes supply neurons with ascorbate in the brain or how the intracellular ascorbate level is established. In addition, the model may have predictive potential for planning effective supportive care during medical and nutritional treatments [34]. It has been demonstrated that ascorbate and its spatial distribution, facilitated by dedicated membrane transporters, are necessary for functioning of the human organism. Therefore, its deficiency may cause serious health consequences ranging from an acute syndrome caused by a severe deficiency to chronic, difficult to follow, deterioration, when the deficiency is mild but persistent [25, 55]. These facts inspire numerous studies on the possible ascorbate medical applications. For many years a large body of clinical and experimental data has been gathered. It has been shown, for example, that ascorbate improves the physical performance as well as intellectual capability [82, 83], strengthens the immunological system [10, 84] or enhances the wound healing processes [85]. Vitamin C has also been shown to synergize with standard chemotherapy in the treatment of both solid and hematopoietic cancer cells and has even been used as an active ingredient in its own rights [1, 34, 86, 87]. Patients with hematopoietic malignancy or other cancers are often markedly vitamin C-deficient, and restoring or maintaining physiological levels has been shown to slow malignant cell growth in multiple settings, including leukemia [88]. High concentrations of ascorbate have been considered as an element for support of cellular therapies especially when stem cell reprograming is required [89, 90]. The ascorbate supplementation is also considered as a measure for slowing down the progression of degenerative processes, especially those leading to neurodegenerative diseases [2, 47, 91].

It is commonly believed that at low doses, vitamin C acts as an antioxidant and maintains sufficient levels of iron in the ferrous state to promote the activity of dioxygenases. By quenching free radicals, vitamin C can therefore protect against mutations induced by oxidative DNA damage, lipid peroxidation, and the oxidation of amino acid residues so as to maintain protein integrity [2]. Maintaining local redox potential by ascorbate is crucial in many metabolic processes, as well as for the formation of metabolic intermediates that are known to play an important role in modulating the activity of epigenetic regulators, such as *a*-ketoglutarate and other citric acid cycle intermediates [70]. At higher concentrations ascorbate can behave as a pro-oxidant, causing oxidative stress and/or depleting glutathione, which leads to the accumulation of ROS, increasing oxidative stress and leading to cell death [92, 93]. The enzyme catalase, under physiological conditions, can metabolize H_2_O_2_. However, elevated basal levels of ROS, deficiency in catalase activity or increased uptake of vitamin C by tumor cells could render them selectively vulnerable to the pro-oxidant effect of high-dose vitamin C, as demonstrated in a number of studies on cultured cells [94–96].

Pharmacokinetic studies in humans have shown that intravenous administration of sodium L-ascorbate can generate up to 30 mM peak plasma levels, 100-fold higher than the levels produced by high-dose oral administration, and is not toxic due to the rapid elimination (a few hours [19, 58]). Recent clinical trials and case studies have shown efficacy of vitamin C as an anticancer agent when administered IV at high doses ranging from 0.4 to 1.5 g ascorbate/kg body weight to treat patients with a variety of solid tumors including breast, ovarian, prostate, kidney, lung, and liver cancer [72, 97–102]. Recently, high-dose vitamin C was shown to be selectively toxic to KRAS or BRAF mutant colorectal cancer cells [93]. Given that high-dose vitamin C can promote increased redox-active iron mobilization and glutathione depletion [93], the ability to induce ferroptosis could be an additional mechanism by which vitamin C can exert its function as an anticancer therapy.

## Conclusion

Ascorbate is a necessary element of physiological and cellular homeostasis. It functions as an antioxidant at low concentrations but at higher concentrations it increases the pool of labile iron ions, accelerating free radical formation. The concentration-dependent mode of action requires the precise maintenance of ascorbate concentration in aqueous compartments so the needed redox potential is generated and maintained. This can be achieved only by a mechanism able to adjust ascorbate quantity in a specific aqueous compartment in the body. The adjustment requires that the ascorbate is transferred across the bordering structure in both directions. At the cellular level, there are two proteins (SVCT1 and SVCT2) specifically transferring ascorbate in the direction of the cell interior. There is no known protein capable of transferring ascorbate in the opposite direction. This causes a serious difficulty in the construction of physiological or cellular models for water-soluble ascorbate homeostasis. Recently, it has been demonstrated that ascorbate is able to diffuse through model lipid bilayers [29, 30]. This discovery provides a missing element for the construction of an effective and coherent conceptual model describing the mechanisms leading to the generation of ascorbate distribution within various body aqueous compartments. The model presented in the paper describes a molecular mechanism responsible for ascorbate distribution at the physiological level. The model accounts for two qualitatively and quantitatively adjustable ascorbate fluxes: the genetically controlled active transport, facilitated by membrane proteins, and passive diffusion through the lipid bilayer. The model is able to resolve most issues related to experimental observations, which could not be explained exclusively based on the action of membrane proteins. According to the model, a local physiological level of ascorbate is controlled directly by the expression of transporting proteins in relevant cells. The model allows one to propose general rules for spatial localization of transporting proteins – specifically, that the SVCT1 transporter is positioned in the plasma membrane of epithelial cells separating the external aqueous phases from the intracellular aqueous phase, whereas the SVCT2 transporter is in the plasma membrane separating the interstitial and intracellular aqueous phases. The spatial arrangement of active transporters supplemented by passive diffusion explains all elements of physiological control of ascorbate homeostasis. The new model, by treating ascorbate as a membrane permeable mono-ion, also offers a prediction regarding the ascorbate distribution within a single cell. Assuming that the cell interior consists of different aqueous phases surrounded by dedicated membranes indicates that these membranes may control the spatial distribution of ascorbate inside a cell. Since ascorbate transporters are not present in endo-membranes, other membrane properties should affect local ascorbate concentration. The passive diffusion of charged compound across the lipid bilayer is sensitive to the membrane electric potential and pH differences. Both parameters are independently controlled by dedicated membrane proteins (such as Na^+^K^+^ATPase and V-ATPase). The dependence allows for rapid adjustment of ascorbate distribution at the cellular level. In summary, the presented model, despite serious simplifications with respect to local water activity, electrostatics of intracellular space and the presence of a large fraction of interfaces, toward which ascorbate has high affinity, has proved to be very effective in explaining many unresolved issues at both physiological and cellular levels [62, 103–105].

## Data Availability

All data generated or analyzed during this study are included in this published article.

## Abbreviations

*a*-KGDDs enzymes: Alpha-ketoglutarate-dependent dioxygenases
ALKBH: AlkB homolog
BRAF: Gene encoding B-raf protein
DHA: Dehydroascorbate
DMT1: Divalent metal transporter 1
FAD: Flavin adenine dinucleotide
GIT: Gastrointestinal tract
GLUT: Glucose transporters
GSH: Glutathione
GULO: L-gulono- *γ*-lactone oxidase
HIF: Hypoxia-inducible factor
JHDM: Jumonji C (JmjC) domain-containing histone demethylases
JmjC: Jumonji C domain
KRAS: Gene encoding K-Ras protein
logP: Logarithm of a partition coefficient
NAD: Nicotinamide adenine dinucleotide
ROS: Reactive oxygen species
SK-MEL-131: Human melanoma cell line
SVCT: Sodium vitamin C transporter
TET: Ten-eleven translocation
TfR1: Transferrin receptor 1
